# Non-alcoholic fatty liver disease and socioeconomic determinants in an Iranian cohort study

**DOI:** 10.1186/s12876-023-02964-4

**Published:** 2023-10-09

**Authors:** Zahra Sadeghianpour, Bahman Cheraghian, Hamid Reza Farshchi, Mohsen Asadi-Lari

**Affiliations:** 1 Department of Epidemiology, School of Public Health, Iran University of Medical Sciences, Tehran, Iran; 2 Department of Biostatistics and Epidemiology, Alimentary Tract Research Center, Clinical Sciences Research Institute, School of Public Health, Ahvaz Jundishapur University of Medical Sciences, Ahvaz, Iran; 3 MRC/ARUK Centre for Musculoskeletal Ageing Research, National Institute for Health Research (NIHR) Nottingham Biomedical Research Centre, Division of Physiology, Pharmacology and Neuroscience, School of Life Sciences, University of Nottingham, Nottingham, NG7 2UH UK; 4 Oncopathology Research Centre, Iran University of Medical Sciences, Tehran, Iran

**Keywords:** Prevalence, Non-alcoholic fatty liver disease, Socioeconomic determinants, Cohort study

## Abstract

**Background:**

Non-alcoholic fatty liver disease (NAFLD) is widespread worldwide. On the other hand, social inequality and socioeconomic status (SES) can affect all aspects of health. Therefore, this study aimed to investigate the relationship between SES indicators and NAFLD.

**Methods:**

This was a cross-sectional study using data from the registration phase of the Hoveyzeh Cohort Study, which included 10,009 individuals aged 35–70 years from May 2016 to August 2018. Fatty liver disease was determined based on Fatty Liver Index (FLI). The crude and adjusted odds ratios were calculated by logistic regression analysis to estimate associations between the fatty liver index and SES after controlling the potential confounders.

**Results:**

According to the FLI index, there were 2,006 people with fatty liver (28%) and 5,246 people without fatty liver (72%). Several 4496 people (62%) were women. The chi-square test showed significant relationships between the educational level and skill level (*P* < 0.001), the wealth index (*P* < 0.001), and Townsend Index (*P* < 0.001) with fatty liver index. In multivariable analysis, after adjustment for age, sex, physical activity, smoking, type of residence, calorie intake, dyslipidemia, skill level, and diabetes, the wealth index (*p* < 0.001) was positively associated with the fatty liver index. Besides, a reverse and significant association was seen between the Townsend index and the fatty liver index(*p* < 0.001). In contrast, no significant associations were seen between gender and educational level with the fatty liver index.

**Conclusions:**

A more vulnerable SES is associated with NAFLD. Fatty liver index and socioeconomic indicators can be powerful monitoring tools to monitor health differences in diagnosing NAFLD.

## Background

In many regions of the world, (NAFLD) is the most prevalent form of liver disease [1]. The prevalence of NAFLD is estimated as high as 25%, and more than 2 billion people are affected by this disease worldwide [2]. In addition, NAFLD is expected to be the leading cause of liver disease-related deaths by 2030 [3] and a leading cause of cirrhosis and hepatocellular carcinoma, for which no approved therapy exists. However, despite NAFLD increasing importance and prevalence, it is not well known by the public, policymakers, and even healthcare providers [1, 2]. NAFLD is the accumulation of excess fat in liver cells that is not due to alcohol consumption. Fatty liver occurs when 5 to 10 percent or more of liver weight is composed of fat (steatosis) (https://liverfoundation.org/for-patients/about-the-liver/diseases-of-the-liver/non-alcoholic-fatty-liver-disease.). Non-alcoholic steatohepatitis (NASH) is the most severe form of NAFLD, affecting 6 million individuals worldwide. It is estimated that 10,000 Iranians suffer from liver failure (cirrhosis) annually, and 5,000 die (https://liverfoundation.org/for-patients/about-the-liver/diseases-of-the-liver/non-alcoholic-fatty-liver-disease.). Even though the evidence base for the investigation and treatment of NAFLD is being established, there is no practical advice to help develop services and provide appropriate care for this disease [4].

While the golden standards for measuring fatty liver are ultrasound and liver biopsy, the measurement based on them is not practical for many large-scale studies [5]. however, NAFLD can be diagnosed using many non-invasive methods including FLI, hepatic steatosis index (HSI), lipid accumulation product (LAP), liver fat score (LFS), hepatorenal ultrasound index (HRI), and regular abdominal ultrasound (AUS) [6]. In addition, there are many studies that prefer the FLI in population-based studies [7, 8], so in the present study, the fatty liver index (FLI) algorithm based on body mass index is is far away. We used waist, serum triglyceride, and gamma-glutamyl transferase, which in previous studies, this index has shown good predictive performance in the diagnosis of NAFLD [5, 9]. According to previous studies, the validity and reliability of this method are acceptable. For the diagnosis of NAFLD, they showed that the sensitivity and specificity were at least 86 and 87% [5]. In addition, the sensitivity and specificity's FLI were evaluated in other studies. In Huang et al.'s study, FLI was shown to diagnose NAFLD with a good AUROC of 0.834 (0.825–0.842). The FLI cut-off point for the diagnosis of NAFLD was 30. The sensitivity was 79.89%, and the specificity was 71.51% in middle-aged and elderly Chinese. This study suggested FLI as a proper non-invasive method to diagnose NAFLD [10]. Subsequently, the sensitivity and specificity's FLI in a Dutch population with a survey of 2652 elderly patients, were 62% and 81%, respectively [9].

Many factors, such as physiological, genetic, environmental, and social factors, play a role in the occurrence of NAFLD. Social factors have always been mentioned as factors related to health and significantly impact the type, size, and distribution of health in societies [11]. Several health-related studies have examined their association with health outcomes [11, 12]. According to some studies, poorer people are most affected by liver disease, making liver disease the main problem of health inequality [13]. A recent epidemiological study has shown that NAFLD is associated with SES in the Iranian population [14]. Also, a cross-sectional study in the Chinese population in 2020 showed that the prevalence of non-alcoholic fatty liver disease gradually increased with increasing income [15]. On the other hand, there is equally little vital evidence to suggest otherwise [16]. SES is determined by education, employment, and wealth. In addition to these three parameters, composite indicators of socioeconomic class are frequently used because of their greater comprehensiveness [16, 17]. However, the reported association between SES and NAFLD is currently controversial. Therefore, this study investigated the association between a more comprehensive SES composite, including educational level and qualification, wealth index, Townsend deprivation index, and NAFLD. Our purpose in this study was to examine the prevalence of NAFLD and its correlation with patients' SES in a relatively large cohort in Hoveyzeh, southwest Iran.

## Methods

### Study design and participants

This cross-sectional study used data from the Hoveyzeh Cohort Study (HCS) registration phase. One of the Persian cohort centers is the Hoveyzeh Cohort Center. The HCS is a prospective population-based cohort study of 10 009 adults (age 35–70 years) recruited from May 2016 to August 2018, designed to assess NCDs in southwest Iran. The Hoveyzeh cohort center is one of Iran's Prospective Epidemiological Research Studies sites (the PERSIAN Cohort Study), including 180 000 Iranian adults. The study population mainly consists of Arabs from urban and rural regions of Hoveyzeh and Sousangerd [18, 19]. The inclusion criteria comprised Iranians residing in Hoveyzeh between the ages of 35 and 70 who were willing to participate in the research. Participants with chronic liver disease (autoimmune hepatitis, hemochromatosis, Wilson disease, hepatitis B and C, and IV), a fatty liver index between 30 and 59, and alcohol consumption were excluded from this analysis.

### Fatty liver index criterion (FLI)

While ultrasound and liver biopsy are the gold standards for measuring fatty liver, these tests are not practical for many large-scale studies; therefore, in the present study, we used the fatty liver index (FLI) algorithm based on body mass index, waistline, serum triglycerides, and gamma-glutamyl transferase; which demonstrated valuable predictive performance in the diagnosis of NAFLD in previous studies [5, 9, 20]. The sensitivity and specificity of this approach for diagnosing NAFLD were at least 86 and 87 percent, respectively, according to earlier research [5].

The FLI is an algorithm based on four variables: Body Mass Index (BMI), Waist Circumference (WC), Serum Triglycerides (TGL), and Gamma Glutamyl Transferase (GGT), which has shown good predictive performance in the diagnosis of NAFLD and corresponds to the following formula:

$$FLI\;=\left(e^{0.953\;\_\;\log e\;\left(triglycerides\right)\;0.139\;\_\;BMI\;0.718\;\_\;\log e\;\left(GGT\right)\;0.053\_waist\;circumference\;\_\;15.745}\right)/_{\left(1+e^{0.\;953\;\_\;\log e\;\left(triglycerides\right)\;139.0\;\_\;BMI\;0.718\;\_\;\log e\;\left(GGT\right)\;0.053\;\_\;waist\;circumference\;15.745}\right)}\;\ast\;100\;$$

The value of the FLI index ranges from 0 to 100. The FLI score of 60–100 is considered to identify fatty liver disease with a sensitivity of 86%. In comparison, the FLI cut-off point < 30 is considered to diagnose fatty liver disease with a specificity of 87%. Standardized regression coefficients indicate that FLI is the most significant predictor of waist circumference, followed by GGT, TG, and BMI [5, 9, 20, 21].

### Measuring Socioeconomic indicators (SES)

We used four indices to analyze SES: the Townsend deprivation index to measure regional deprivation, the wealth index as a household-level index, and educational attainment and skill level as individual-level socioeconomic indicators. In this study, educational attainment is determined by the number of years of schooling without failure and the years a person has attended school or college. Consistently mentioned as elements connected to health, social factors substantially affect the kind, magnitude, and distribution of health in societies [11]. In this study, we used composite indicators that group different socioeconomic domains on a quantitative scale; in this way, the components of each domain are weighted in a certain way, and from the result, a rank is obtained for each person, indicating the person's economic-social status. Among the combined indices, the wealth and Townsend deprivation indexes are noteworthy [16].

### Measuring wealth index

The wealth index was calculated using the following nine assets: washing machine, computer, vacuum cleaner, freezer, motorcycle, car ownership, home ownership, access to the internet, and number of people per room. Each asset was included in the study as a variable with two states. First, the correlation values between each of the variables mentioned above were calculated separately in the form of a matrix; then, based on the calculated correlation values, a coefficient was assigned to each variable, representing the different weights of each variable in determining the relevant index. By multiplying each of these coefficients by a variable value (i.e., zero or one) and summing all the resulting values, a total score was obtained for each household. Based on these scores, each household was classified into one of five groups: the poorest, poor, average, rich, and the wealthiest, according to the percentiles of the distribution of scores [17].

### Measuring townsend deprivation index

The Townsend Deprivation Index was used to determine the level of deprivation in the region [22]. The Townsend deprivation index is a measure of material deprivation first introduced by Peter Townsend in 1987 [23]. A Townsend score can be calculated using a combination of four census variables for any geographical area, provided census data is available for that area. Four variables were used to calculate this index: the percentage of unemployed (between 16 and 64 years old), the percentage of households without a car, the percentage of households that do not own a house, and the percentage of households with a high population density (more than one person per room), and the steps to determine this index are as follows:

1- Calculating the relative frequency of each of the four variables above (as a proportion, not a percentage) 2- For the two indicators, unemployment, and population density, all numbers were first added by one. Then the natural logarithm of them was formed. 3- The average and standard deviation were calculated for each of the four variables. 4- The standard values (Z-score) were calculated for each of the four variables. 5- The standard values of all four variables above were added, and then a general value was obtained for each region as a Townsend score; the higher this value was, the higher the degree of deprivation in the region [24].

### Measuring skill level

International Standard Classification of Occupations (ISCO-08), with a four-level structure that allows all occupations worldwide to be categorized into 436-unit groupings, was used. This is the most specific classification system level, and all occupations are divided into four broad groups according to skill level and required expertise. One skill level covers the most basic positions and manual duties. Those whose jobs required essential hand tools were assigned to this level. For almost all tasks requiring a skill level of 2, understanding information such as safety instructions, maintaining written records of completed work, and executing basic arithmetic calculations effectively was needed. Jobs at skill level 3 required advanced abilities and specialized knowledge. In addition, level 4 positions often require solving complex problems and making decisions based on a broad range of theoretical and experimental knowledge in a particular area. At this level, managers and technical officers were assigned [25, 26].

### Anthropometric measurements

Anthropometric measurements were performed by trained personnel. Height (cm) was measured using a stadiometer (Seca 206) while standing without shoes, shoulders relaxed, facing forward, head and back facing the wall. Body weight (kg) was measured on a stand scale (Seca 755) while wearing light clothing. A locking tape measure (Seca) was also used to measure waist, wrist, and hip circumference (cm).

### Biomedical measurements

Participants fasted for approximately 10 to 12 h on the day of enrollment. Each participant drew 27 ml of blood. Based on The ATP III (Third Report of the National Cholesterol Education Program Expert Panel on Detection, Evaluation, and Treatment of High Blood Cholesterol in Adults) defines dyslipidemia as any abnormality of lipoprotein metabolism, including having at least one of the following: TC ≥ 200 mg/dL; TG: ≥ 150 mg/dL; LDL-C ≥ 130 mg/dL and HDL-C < 40 mg/dL [27]. Diabetes is defined as fasting blood glucose ≥ 126 mg/dl, taking blood glucose-lowering medications, or self-reported diabetes diagnosed by a physician [28].

### Food intake measurements

In this study, Nutritional status was assessed using the validated national Iranian Food Frequency Questionnaire (FFQ) [29–31].the Frequency food questionnaire (FFQ) was completed One-year food frequency. Then Micronutrient and macronutrient intakes are reported by N4 nutrition analysis software.

### Statistical analysis

The variables to be studied were first defined using descriptive statistics techniques, such as frequency tables, graphs, and indices of central tendency and appropriate dispersion to examine the data. The Kolmogorov–Smirnov test was then used to determine the normality of the distribution of the quantitative variables. Principal component analysis (PCA) was used to calculate the wealth index based on household wealth. The chi-square or Fisher's exact test determined the association between sex and education, age groups, residence, BMI, daily activity level, smoking status, daily energy intake, and diabetes and dyslipidemia with fatty liver disease. In addition, an independent t-test comparing waist circumference, dyslipidemia, and GGT levels between two groups of patients and healthy individuals, and a one-way analysis of variance evaluating mean fatty liver index scores between more than two groups, including wealth status, regional deprivation, skill level, and education, were performed. An unconditional logistic regression analysis with odds ratios and confidence intervals was performed to account for confounding variables in the association between SES and NAFLD. The significance threshold of the tests was deemed to be below 0.05. SPSS 26.0 was used for data analysis, whereas Stata 16.0 was utilized for principal component analysis (PCA).

## Results

Among 10,009 participants, eight individuals were excluded from the study because of the diseases listed in the exclusion criteria and alcohol consumption. In addition, 2,749 individuals were 30–59 for the fatty liver index, so they were excluded from the investigation according to the exclusion criteria. Finally, 7,252 individuals remained in the research. The individuals' mean and standard deviation age were 48.7 ± 9.09 years, ranging between 35 and 70 years, 62% (4496) were women, 62% resided in urban areas and 64% were illiterate. Demographic and clinical features of participants with fatty liver disease are shown in Table 1.

**Table 1:** Demographic and clinical characteristics of the studied subjects based on fatty liver disease

Variable		Total	FLI -** N (%)	FLI + *** N (%)	*P*-value *
**Age (years)**	**35–44**	2816	856 (30.4)	1960 (69.6)	< 0.001
	**45–59**	3381	803 (23.8)	2578 (76.2)	
	** ≥ 60**	1055	347 (32.9)	708 (67.1)	
**Gender**	**Male**	2758	795 (28.8)	1963 (71.2)	0.083
	**Female**	4494	1211 (26.9)	3283 (73.1)	
**Area**	**Urban**	4492	1010 (22.5)	3482 (77.5)	< 0.001
	**Rural**	2760	996 (36)	1764 (64)	
**Smoking**	**No**	5861	1542 (26.3)	4319 (73.7)	** < 0.001**
	**Yes**	1391	464 (33.4)	927 (66.6)	
**BMI**	**Underweight**	147	147 (100)	0	< 0.001
	**Normal**	1556	94 (6)	1462 (94)	
	**Overweight**	1970	388 (19.7)	1582 (80.3)	
	**Obese**	9357	9 (0.3)	3570 (99.7)	
**Physical activity (*MET* Score)**	**Q1**	1868	414 (22)	1454 (78)	< 0.001
	**Q2**	1807	432 (24)	1375 (76)	
	**Q3**	1831	565 (31)	1266 (69)	
	**Q4**	1746	595 (34)	1151 (64)	
**Energy intake (Kcalories per day)**	**Q1**	1814	551 (30.4)	1263 (69.6)	0.001
	**Q2**	1812	529 (29.2)	1283 (70.8)	
	**Q3**	1813	471 (26)	1342 (74)	
	**Q4**	1813	455 (25.1)	1358 (74.9)	
**Dyslipidemia**	**Normal**	3938	1579 (40.1)	2359 (59.9)	< 0.001
	**High**	3314	427 (12.9)	2887 (87.1)	
**Diabetic**	**No**	5552	1784 (32.1)	3768 (67.9)	< 0.001
	**Yes**	1700	222 (13.1)	1478 (86.9)	
**Educational level**	**Illiterate**	4611	1346 (29.2)	3265 (70.8)	< 0.001
	**Primary school**	1164	281 (24.1)	883 (75.9)	
	**Secondary school**	474	110 (23.2)	364 (76.8)	
	**High school**	513	146 (28.5)	367 (71.5)	
	**University**	490	123 (25)	367 (75)	

^*^P-value in the chi-square test

^**^FLI-: A negative FLI index is someone who does not have fatty liver (Fatty liver index < 30)

^***^ FLI + : A positive FLI index is someone with fatty liver (Fatty liver index:60–100)

According to the FLI index, 2,006 individuals with fatty liver (28%) and 5,246 individuals without fatty liver (72%). The results showed that 927 (66.6%) of smokers had fatty liver, while the prevalence of fatty liver in nonsmokers was 73.7%. After adjusting for age, gender, and smoking status in multivariate regression, smoking was a protective factor for smokers (OR = 0.7, CI95% = 0.61–0.79, *P* < 0.001). The average BMI of the individuals was 29.57 ± 5.83. One thousand four hundred fifty-four people (78%) with the lowest physical activity suffered from fatty liver. The mean value of GGT and TG was 28.07 (U/L) and 172.2 (mg/dl), respectively. One thousand seven hundred participants (23%) had diabetes (FBS > 126), of whom 1478 subjects (86%) had fatty liver. According to the chi-square test results, there was a significant association between the variables age, type of residence, level of education, BMI, dyslipidemia, diabetes (*P* < 0.001), and fatty liver(Table 1).

Regarding the wealth index, 877 persons (approximately 62%) of the poorest class suffered from fatty liver, while 1181 persons (81%) of the wealthiest class suffered from fatty liver. More than 80% of individuals in skill level 3 were affected by fatty liver. When the association between the Townsend index and fatty liver disease was examined, it was found that more than 80 percent of the wealthiest class belonged to the affected group. The results of the chi-square test showed that there was a significant relationship between skill level (*P* < 0.001), wealth index (*P* < 0.001), and regional deprivation index (*P* < 0.001) with fatty liver (Table 2).

**Table 2:** Socioeconomic indicators of the studied people based on fatty liver index

**Variable**		Total	FLI -** N (%)	FLI + *** N (%)	*P*-value *
**Wealth Status**	**Poorest**	1433 (100)	556 (38.8)	877 (61.2)	< 0.001
	**Poor**	1445 (100)	489 (33.8)	956 (66.2)	
	**Moderate**	1442 (100)	376 (26.1)	1066 (73.9)	
	**Rich**	1492 (100)	326 (21.8)	1166 (78.2)	
	**Richest**	1440 (100)	259 (18)	1181 (82)	
**Skill Level**	**Skill Level I**	221 (100)	91 (41.2)	130 58.8))	< 0.001
	**Skill Level II**	1625 (100)	452 (27.8)	1173 (72.2)	
	**Skill Level III**	90 (100)	17 (18.9)	73 (81.1)	
	**Skill Level IV**	304 (100)	72 (23.7)	232 (76.3)	
**(Townsend deprivation index)**	**Most Affluent**	1802 (100)	336 (18.6)	1466 (81.4)	< 0.001
	**Affluent**	1351 (100)	324 (24)	1027 (76)	
	**Moderate**	1356 (100)	386 (28.5)	970 (71.5)	
	**Deprived**	910 (100)	326 (35.8)	584 (64.2)	
	**Most Deprived**	1833 (100)	634 (34.6)	1199 (65.4)	

^*^P-value in the chi-square test

^**^FLI-:A negative FLI index is someone who does not have fatty liver (Fatty liver index < 30)

^***^ FLI + positive FLI index is someone with fatty liver (Fatty liver index:60–100)

Univariate logistic regression models were used to assess the strength of associations between various factors and fatty liver disease, which showed a significant association between FLI and wealth index (OR = 2.89 (CI 95% 2.43–3.43), *P* < 0.001), indicating that developing fatty liver was 2.9 times higher in the wealthiest class than in the lowest. Concerning the Townsend index, these findings revealed that the likelihood of suffering from fatty liver disease was 2.3 times more among the wealthiest class than the most disadvantaged class (reference class) (OR = 2.3 (CI 95% 0.61–0.86), *P* < 0.001).

In addition, there was a significant association between skill level and fatty liver disease; the likelihood of suffering from fatty liver disease was 81% greater for those with skill level two compared to those with skill level one (OR = 1.81 (CI 95% 1.36–2.42), *P* < 0.001). Those with secondary education were 2.3 times more likely to have fatty liver disease compared to illiterate people (OR = 2.3 (CI 95%: 0.61–0.86), *P* < 0.001). No correlation was identified between gender and the disease. The odds of fatty liver disease were most significant among those aged 45 to 59, 40 percent higher than the reference group (44–35). People living in urban areas were 94% more likely to have fatty liver than those living in rural areas (OR = 1.94 (CI 95%: 1.75 to 2.16), *P* < 0.001). Fatty liver disease was independently related to physical activity, diabetes, dyslipidemia, and caloric intake (Table 3).

**Table 3:** Crude and adjusted odds ratios using the univariate logistic regression model and multiple regression model

**Variable**		**Crude ORs** **(CI 95%)**	*P*-value*	**Adjusted ORs** **(CI 95%)**	*P*-value **
**Age(yesrs)**	35–44	1	< 0.001	1	0.002
	45–59	1.40 (1.25– 1.57)		1.34 (1.07–1.68)	
	≥ 60	0.89 (0.76– 1.03)		0.75(0.51–1.11)	
**Gender**	Male	1	0.083	1	0.32
	Female	1.09 (0.98 – 1.22)		1.20 (0.83– 1.74)	
**Area**	Rural	1	< 0.001	1	0.002
	Urban	1.94(1.75–2.16)		1.43(1.13–1.81)	
**Smoking**	No	1	< 0.001	1	< 0.001
	Yes	0.71(0.62–0.80)		0.63(0.50–0.79)	
**Physical activity (*MET* Score)**	Q1	1.81(1.56–2.10)	< 0.001	1	0.002
	Q2	1.64(1.42–1.90)		0.79(0.55–1.15)	
	Q3	1.15(1.007–1.33		0.57(0.39–0.81)	
	Q4	1		0.57(0.41–0.79)	
**Energy intake (Kcalories per day)**	Q1	1	0.001	1	< 0.001
	Q2	1.05(0.91–1.22)		1.68(1.14–2.48)	
	Q3	1.24(1.07–1.43)		1.83(1.27–2.65)	
	Q4	1.30(1.12–1.50)		3.00(2.09–4.32)	
**Dyslipidemia**	normal	1	< 0.001	1	< 0.001
	High	4.52(4.01–5.1)		6.3(5.05–7.8)	
**Diabetes**	No	1	< 0.001	1	< 0.001
	Yes	3.15(2.7–3.66)		2.51 (1.83–3.45)	
**Educational level**	Illiterate	1	0.001	1	0.59
	Primary school	1.29(1.11–1.50)		1.10(0.82–1.49)	
	Middle school	1.36(1.09–1.70)		1.26(0.87–1.84)	
	High school	1.03(0.84–1.26)		0.93(0.64–1.35)	
	University	1.23(0.99–1.52)		0.90(0.55–1.48)	
**Wealth Status**	Poorest	1	< 0.001	1	< 0.001
	Poor	1.23 (1.06–1.44)		1.35(0.94–1.92)	
	Moderate	1.79 (1.53–2.10)		1.42(0.99–2.02)	
	Rich	2.26 (1.92–2.66)		2.17(1.51–3.11)	
	Richest	2.89 (2.43–3.43)		2.54(1.73–3.74)	
**Skill Level**	Skill Level I	1	< 0.001	1	0.03
	Skill Level II	1.81(1.36–2.42)		1.62(1.16–2.27)	
	Skill Level III	3.00(1.66–5.43)		2.06(1.02–4.14)	
	Skill Level IV	2.25(1.54–3.28)		1.67(0.93–2.96)	
**(Townsend deprivation index)**	Most Affluent	2.3(0.61–0.86)	< 0.001	1.60(1.15–2.23)	< 0.001
	Affluent	1.67(1.43–1.96)		1.39(0.97–1.99)	
	Moderate	1.32(1.14–1.54)		1.73(1.32–2.28)	
	Deprived	0.94(0.80–1.11)		1.63(1.04–2.55)	
	Most Deprived	1		1	

^*^P-value in the univariate logistic regression model

^**^P-value in the multiple logistic regression model

Multivariate logistic regression analysis was used to control confounding variables. All variables with a significance level (*p* < 0.2) in univariate logistic regression were included in the model and reported as odds ratios. By adjusting age, sex, residence type, smoking, physical activity, energy, dyslipidemia, debate, educational level, Wealth Status, Skill Level, Townsend index, multiple logistic regression revealed that age, type of residence, smoking status, physical activity, calorie intake, dyslipidemia, diabetes, and skill level had a significant relationship, although education level and gender, and fatty liver disease were not statistically significant. In addition, the wealthiest group had 2.5 times the risk of acquiring the illness compared to the poorest (reference) group (`OR = 2.54 (CI 95% confidence interval: 1.73–3.47), *P* < 0.001). Also, people with skill level 2 had 62% higher odds of developing fatty liver (`OR = 1.62 (CI 95% confidence interval: 1.16–2.27), *P* = 0.03), while people with skill level 3 were 2 times more(reference: Skill 1)(`OR = 2.06 (CI 95% confidence interval: 1.02–4.14), *P* = 0.03). Regarding the Townsend index, these findings revealed that those in the most affluent class were 60% more likely to develop NAFLD than those in the most disadvantaged class (OR = 1.60 (CI 95% confidence interval: 1.15–2.23), *P* < 0.001). In terms of age group, the odds of NAFLD in people aged 45–59 years was 34% higher than that of people between 35–44 years old (OR = 1.34(CI 95% confidence interval: 1.07–1.68), *P*0.002 =). People living in urban areas were 43% more likely to have NAFLD than people living in rural areas (OR = 1.43(CI 95% confidence interval: 1.13–1.81), *P* = 0.002 =). Compared to the group with less activity (reference), the risk of NAFLD was 57% less among those with high activity levels. In addition, persons with a higher daily caloric intake had a 3 times greater risk of developing the condition than those with a lower calorie intake. (OR = 3.00 (CI 95% confidence interval: 2.09–4.32), *P* < 0.001).

People with dyslipidemia were 6.3 times more likely to suffer from NAFLD than those with normal levels (OR = 6.3 (CI 95% confidence interval 5.05–7.8), *P* < 0.001). In those with diabetes, the risk of NAFLD was 2.5 times more than in those without diabetes (OR = 2.51 (CI 95% confidence interval: 1.83–3.45), *P* < 0.001). The likelihood of NAFLD was 37% lower among smokers compared to nonsmokers (reference) (OR = 0.63 (CI 95% confidence interval 0.59–0.79), *P* < 0.001) (Table 3).

**Fig. 1:**
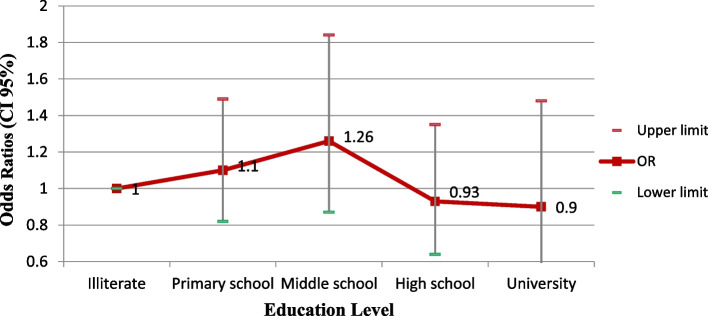
Adjusted odds ratios (CI 95%)of the fatty liver index by education level

At the level of education, Primary school and high school groups showed the highest odds of infection but did not show a significant relationship (Fig. 1).

**Fig. 2:**
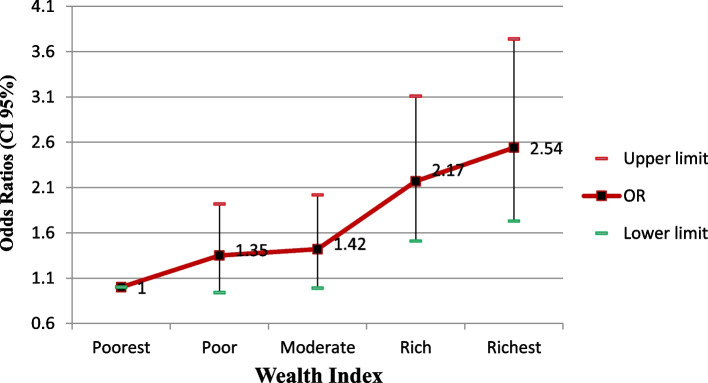
Adjusted odds ratios (CI 95%)of the fatty liver index by wealth index

According to Fig. 2, a direct and significant relationship was observed between the wealth index and the odds of fatty liver disease (Fig. 2).

**Fig. 3:**
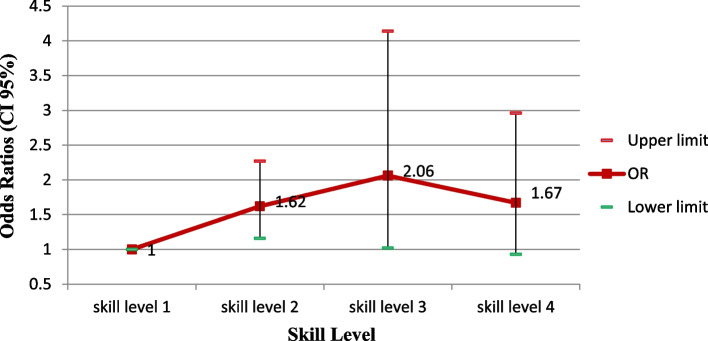
Adjusted odds ratios (CI 95%) of the fatty liver index by skill level

There was a significant relationship between skill level and fatty liver disease. According to Fig. 3, skill level 1 and 2 shows the highest odds of contracting the disease (Fig. 3).

**Fig. 4:**
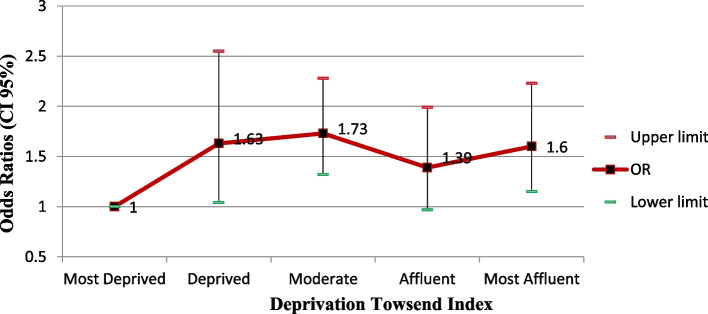
Adjusted odds ratios (CI 95%) of the fatty liver index based on the Deprivation Towsend index

According to Fig. 4, the Townsend index shows a direct and significant relationship(Fig. 4).

## Discussions

This is the first report of NAFLD from the Hoveyzeh Cohort Study, which examined data from 10,009 individuals with a mean age (SD) of 48.7 (± 9.09) years who were eligible to enter this research. We used the Fatty Liver Index for NAFLD [5, 9]. In a study by Lind et al., FLI was preferred in a population-based setting, while LFS performed best in a high-risk setting [8]. In a study aimed at validating and comparing eight models related to NAFLD that were developed by simple indices and their cut-off values in the Chinese population, it was shown that FLI can be one of the most accurate and applicable models among the eight models for non-invasive diagnosis of NAFLD in Both groups are male and female [7]. Overall, 5,246 patients (52.4%) of the total study population had NAFLD (FLI ≥ 60) There was no significant relationship between gender and FLI (*P* = 0.083). Meanwhile, other studies showed different results. In several studies, men constituted the majority of patients with fatty liver disease [32–34]. This is probably attributed to differences in study design and subjects' ethnicities. This study revealed that FLI has strong associations with several variables, including BMI, where the prevalence of fatty liver increased in higher BMI groups. In addition, this increasing trend was observed for the daily caloric intake index. Several studies showed that obesity is associated with the progression of hepatic fibrosis and worse prognosis (NASH) [20, 35, 36].

Research by Peta et al. on 225 patients with NASH demonstrates that obesity was associated with more advanced hepatic fibrosis [37]. Another study by Koo et al. on 309 Subjects shows that in obese patients, a strong correlation had observed between NAFLD and obesity [38]. Although the research by Koehler et al. showed that Asians have a low body mass index compared to other ethnic populations, with a surprisingly high prevalence of fatty liver disease [39], probably due to the high prevalence of metabolic syndrome in the Asian population [40]. In addition, in a genome-wide study, one of the first genes associated with NAFLD was patatin-like phospholipase domain-containing protein 3 (PNPLA3). Several Asian studies have confirmed this association [41–45].

Dyslipidemia was associated with a significant risk of NAFLD (*P* < 0.001). Karimi et al. reported similar evidence for the association between dyslipidemia and FLI [46]. Our results showed that people with diabetes were more prone to FLI than non-diabetic people. Several large meta-analyses demonstrate that diabetes increases the risk of developing liver diseases [47, 48]. A meta-analysis of 19 observational studies with 296,439 subjects showed that subjects with NAFLD were at a higher risk of having diabetes [48]. A cohort study on 12,853 South Koreans showed that the odds of diabetes was higher in people with NAFLD [49].

Our research examined four important SES variables with fatty liver index. Univariate logistic regression results showed a significant relationship between education level and fatty liver disease. The majority of the Hovyzeh cohort population had a low level of education. In univariate regression, people with secondary education were more likely to suffer from NAFLD; however, after controlling for confounding factors, this relationship was not significant, which is consistent with other studies [32]. A study in India showed no significant relationship between fatty liver disease and education level [50].

Our findings revealed a substantial direct association between the wealth index and the FLI, even after controlling for confounding variables, where people in the highest wealth quintiles were significantly more likely to have fatty liver disease compared to those in the lower wealth quintiles. Other studies have controversially reported the relationship between wealth and fatty liver disease. Our findings may be due to dietary intake and less physical activity of people of the wealthiest group due to non-manual work. A study on the Chinese population also showed that the prevalence of NAFLD increased with the increase in their income [15], while in another study the Korean population with low income and education were more likely to suffer from NAFLD than people with high income and education. For the SES, they used a composite score with income and education ranging from 0 to 100. A significant relationship between income and education was observed (*p* < 0.001). In addition, the results showed that the odds of having NAFLD was significant by an increase of one point in SES, middle-SES, and high-SES [34].

The analysis of this research showed a significant and direct relationship between the Townsend index of deprivation and FLI, where the most affluent people had more odds of suffering from NAFLD compared to the most deprived class. These results can be related to the fact that people who live in more affluent areas have different lifestyles, are less manual workers or in jobs with less physical activity, such as managers and specialists, and therefore, are more exposed to the risk of fatty liver, which is consistent with other studies. In a study by Laitinen et al., children who lived in more deprived areas had higher odds of developing NAFLD in adulthood [51].

In another study of more than half a million participants from the UK Biobank to predict the risk of NAFLD, it was found that people with a lower SES based on the Townsend index were at a higher risk [52]. In addition, place of residence (urban or rural) was associated with fatty liver disease, similar to other studies [34, 53].

The main strength of our study is the large sample size. This results in more accurate estimates because it can be seen in the narrow confidence intervals for the estimated rates. In addition, the utilization of skilled interviewers and the presence of many supervisors are potential benefits of this study. Significantly, we used a diagnostic definition for fatty liver in reliable cohort studies. The sensitivity and specificity of the FLI were assessed in other studies too. Compared with the gold standard (ultrasonography), in an Italian population of 5780 (Modena, Italy), the sensitivity and specificity of FLI were 61% and 86% [5], and in a Dutch population of 2652 elderly patients [9], these figures were, 62% and 81% respectively. As FLI in this study was assessed based on the individuals' self-reporting, specificity was 98%. Still, sensitivity was quite low at 10%, which means that patients with NAFLD were unaware of their illness. A population-based study in northern Iran showed that FLI was a stronger predictor than other measurements for new cases of NAFLD in men and women after seven years of follow-up [53].

This study had limitations too. Lack of clinical investigations, incomplete information on household income and expenditures which is somehow a preferable indicator for economic disaggregation, and other clinical outcome variables which require more duration over time, are part of the limitations in this report.

## Conclusion

Economic and social status is highly associated with NAFLD and the fatty liver index. The association between the wealth index and fatty liver disease index was the strongest among the four assessed indicators. Also, results showed a significant and direct relationship between the Townsend deprivation and skill level index and NAFLD. At the same time, the level of education was not a good predictor of NAFLD. In addition, middle age, inadequate physical activity, diabetes, and dyslipidemia were predictors of FLI.

Most risk factors for NAFLD are controllable; consequently, public health programs can play more important roles. Health professionals and other stakeholders should be aware that these results prevent and better manage fatty liver disease in the population and inform at-risk individuals and patients with a high FLI about additional risk factors and, consequently, a higher risk of developing other illnesses. Long-term disease management, including a healthy lifestyle, is crucial for the prevention and treatment of NAFLD. This may be accomplished by coordinating the integrated programs within primary healthcare services complementing with appropriate social services. 

### Limitations

This study had limitations too. Lack of clinical investigations, which is somehow a preferable indicator for economic disaggregation, and other clinical outcome variables which require more duration over time are part of the limitations in this report. Some confounding factors, including genetic factors, are not adjusted.

## Data Availability

The datasets used and analyzed during the current study are available from the corresponding author upon reasonable request.

## Abbreviations

FLI: Fatty Liver Index
HCS: Hoveyzeh Cohort Study
NAFLD: Non-alcoholic fatty liver disease
SES: Socioeconomic status
NASH: Non-alcoholic steatohepatitis
BMI: Body Mass Index
WC: Waist Circumference
TG: Serum Triglycerides
GGT: Gamma Glutamyl Transferase
ISCO: International Standard Classification of Occupations
PCA: Principal component analysis
